# Use of Empirical Correlations to Determine Solvent Effects in the Solvolysis of *S*-Methyl Chlorothioformate

**DOI:** 10.3390/ijms11052253

**Published:** 2010-05-25

**Authors:** Malcolm J. D’Souza, Stefan M. Hailey, Dennis N. Kevill

**Affiliations:** 1 Department of Chemistry, Wesley College, 120 N. State Street, Dover, Delaware 19901-3875, USA; 2 Department of Chemistry and Biochemistry, Northern Illinois University, DeKalb, Illinois 60115-2862, USA

**Keywords:** solvolysis, nucleophilicity, ionizing power, *S*-methyl chlorothioformate, chloroformates, thioesters, thiochloroformate, Grunwald-Winstein Equation, Linear Free Energy Relationships

## Abstract

The specific rates of solvolysis of *S*-methyl chlorothioformate (MeSCOCl) are analyzed in 20 solvents of widely varying nucleophilicity and ionizing power at 25.0 °C using the extended Grunwald-Winstein Equation. A stepwise S_N_1 (D_N_ + A_N_) mechanism is proposed in the more ionizing solvents including six aqueous fluoroalcohols. In these solvents, a large sensitivity value of 0.79 towards changes in solvent nucleophilicity (*l*) is indicative of profound rearside nucleophilic solvation of the developing carbocation. In twelve of the more nucleophilic pure alchohols and aqueous solutions, the sensitivities obtained for solvent nucleophilicity (*l*) and solvent ionizing power (*m*) are similar to those found in acyl chlorides where an association-dissociation (A_N_ + D_N_) mechanism is believed to be operative.

## 1. Introduction

Six decades ago, Grunwald and Winstein proposed the simple linear free energy relationship (LFER) [1] to correlate the specific rates of solvolysis of initially neutral substrates reacting by an ionization (S_N_1 + E1) mechanism. In the simple Grunwald-Winstein Equation (Equation 1), *k* and *k_o_* are the specific rates of solvolysis in a given solvent and in the standard solvent (80% ethanol), respectively, *c* is a constant (residual) term, and *m* represents the sensitivity to changes in the solvent ionizing power *Y* (initially set at unity for *tert*-butyl chloride solvolyses) [1]. For a leaving group X, it was realized [2,3] that this requirement is closely satisfied by adamantyl derivatives RX (where R = 1- or 2-adamantyl) and a series of *Y_X_* scales are now available [4]. For bimolecular nucleophilically solvent-assisted (S_N_2 and/or E2) reactions, this correlation was later extended (Equation 2) by Grunwald, Winstein, and Jones [5] to include a term governed by the sensitivity *l* to changes in solvent nucleophilicity (*N*). Based on the reasonable assumption that primary methyl substrates in solvolysis reactions would be subject to intense nucleophilic solvent assistance, Schadt, Bentley, and Schleyer [6] used methyl *p*-toluenesulfonate, to arrive at the *N_OTs_* scale. Kevill and Anderson proposed a *N_T_* scale based on the solvolyses of *S*-methyldibenzothiophenium ion [7] in which the leaving group is a neutral molecule, which is little influenced by solvent change, and these values [7,8] have become the recognized standards for considerations of solvent nucleophilicity. In spite of the cautionary comments regarding the use of multiparameter Equations due to the strong covariances observed between the *N* and *Y* values [9], the benefits gained from the application of Equations 1 and 2 to substrates with localized charges [10] have been immensely useful and accurate in predicting reaction pathways in such correlation type analysis. However, dispersions were often observed [11,12] in Grunwald-Winstein correlations when resonance delocalization was possible between the reaction site and an adjacent π-system. Additionally, scatter in Equations 1 and 2 was heightened for solvolyses of α-haloalkyl aryl compounds that proceed via anchimeric assistance (*k*_Δ_) [13]. In consideration of a number of factors, Kevill, and D’Souza proposed a solution [10,14–25] by adding an additional term, the aromatic ring parameter *I* to Equations 1 and 2. In Equations 3 and 4, *h* represents the sensitivity of solvolyses to changes in the aromatic ring parameter *I*. Hence, the magnitudes of the *l*, *m*, and *h* values can give important indications regarding the mechanism of solvolysis [10,14–25]. Two years ago, Martins *et al*. after evaluating their results obtained on using the *hI* term for substrates that (mostly) lacked π-electrons, suggested that the *I* scale may indeed include a nucleophilic component [26]. In a recent review [27] of 30 highly hindered substrates not having appropriately placed π-electrons, we determined that in such substrates the sometimes positive and sometimes negative *h* values that Martins observed [26] is an artifact resulting from the multicollinearity that is present between the *I* values and a linear combination of *N*_T_ and *Y*_X_ values [27]. We have also just demonstrated that the *I* scale is very useful in studies of the solvolyses of compounds containing a double bond in the vicinity of any developing carbocation [28].

(1) $$\text{log}\;(k/k_{o})=mY+c$$

(2) $$\text{log}\;(k/k_{o})=lN+mY+c$$

(3) $$\text{log}\;(k/k_{o})=\text{mY}+hI+c$$

(4) $$\text{log}\;(k/k_{o})=lN+mY+hI+c$$

**Figure 1.**
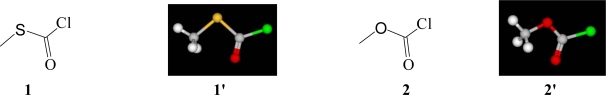
Molecular structures of *S*-methyl chlorothioformate (**1**), and methyl chloroformate (**2**). 3-D images for the *syn* conformer of *S*-methyl chlorothioformate (**1′**), and methyl chloroformate (**2′**).

Thioesters are important biological molecules [29] that can undergo hydrolysis or additional molecular interactions to afford the desired thiol functionality. Furthermore, a “one-pot” synthesis method for *S*-methyl thioesters has been developed by reacting *S*-methyl chlorothioformate (MeSCOCl, **1**) with carboxylic acids [30] to build combinatorial libraries to search for new commercial flavors and/or identification of characteristic flavors in foods [31]. To gain a better understanding of the relationship between the reactivity of thioesters and their structural conformations, Queen *et al*. [32] made a comparison of the dipole moments of a series of aryl and alkyl thiochloroformate esters to their chloro- and fluoroformate analogs in the non-polar solvent benzene. They concluded [32] that such thio- and halo-formate esters prefer a configuration where the halogen atom is in a *cis* position with respect to the alkyl group. Based on observations of the relationship between the atomic charges and dipole moments calculated by the semi-empirical CNDO/2 molecular orbital method, Lee [33] proposed that the alkyl chloro-, thiochloro-, thiofluoro-, thiono-, and dithio-formate esters prefer a configuration that brings the halogen into a *trans* position with respect to the alkyl group. More recently a number of groups [34–41] using a variety of computational studies, experimental techniques, and crystal structure analysis have confirmed Lee’s original proposal [33] that the most stable geometric structures and conformations of the chloroformate (ROCOCl), thiochloroformate (RSCOCl), thiofluoroformate (ROCOF), chlorothionoformate (ROCSCl), and dithiochloroformate (RSCSCl) esters exist in a configuration where the C=O or C=S is *syn* with respect to the alkyl or aryl moiety; *i.e.*, the halogen atom is in a *trans* position with respect to the alkyl or aryl group. In Figure 1, the *syn* conformer of MeSCOCl (**1**) is shown as **1′**, whereas that of methyl chloroformate (MeOCOCl, **2**) is shown as **2′**.

A number of groups have now offered the conclusive explanation [32,33,43–47] that due to the increased initial ground-state resonance stabilization ROCOCl, RSCOCl, ROCSCl, and RSCSCl undergo solvolytic reactions much more slowly than other acid chlorides (RCOCl). On analyzing the rate data for a series of alkyl chloro-, thio-, thiono-, and dithio-formate esters, Queen *et al* proposed [44,45] that the thio- containing substrates hydrolyze by a unimolecular process, whereas their chloroformate counterparts tend to favor a bimolecular mechanism. Lee *et al*. [48,49] studied the solvolysis of **2** and its thio- analogs in a variety of aqueous ethanol, methanol, acetone, and acetonitrile mixtures and concluded that the dithio MeSCSCl underwent unimolecular solvolysis in all solvents, whereas the solvolyses of **2** followed a S_N_2 process. Additionally the authors proposed competing channels for MeSCOCl and MeOCSCl that depended on the ionizing ability of the solvent; with a S_N_2 mechanism favored in ethanol rich mixtures that gradually switched over to a S_N_1 process in the water-rich regions. Lee’s group also advocated [50] for a concerted process for the aminolysis of aryl chlorothionoformates with aniline in acetonitrile. Castro, Santos and co-workers [51–56] followed the kinetics of several thiol, dithio, and thiono analogs of carboxylic acids with different nucleophiles spectrophotometrically. Their results [51–56] are consistent with a stepwise scheme which involves a kinetically significant proton transfer from a zwitterionic to a thermodynamically favorable anionic tetrahedral intermediate.

In an advancing assessment into the practicality of Equations 1–4 for studies of solvolytic mechanisms [10,27,57], we have previously demonstrated [58–61] that there is a side-by-side operation of the association-dissociation (A_N_ + D_N_) and ionization (S_N_1) pathways in the ethyl- and phenyl-thio, and phenyl-thiono analogs of carboxylic acid esters. We also showed that the phenyl dithio ester PhSCSCl, progresses at one extreme with strong nucleophilic solvation of a resonance stabilized carbocation [60,61], whereas, phenyl chloroformate (PhOCOCl), solvolyses by an addition-elimination (A_N_ + D_N_) pathway with the addition step being rate-determining [61,62]. Koo, Lee, and coworkers proposed the presence of a through conjugation of the ring π system with the reaction center in phenyl chlorothionoformate (PhOCSCl) [63], and PhSCSCl [64]. This opinion [63] for PhOCSCl was negated [61], as no evidence was found requiring inclusion of the *h* parameter for ionization reactions with PhOCSCl. For PhSCSCl [61], the large *l* values of 0.69 (Equation 2) or 0.80 (Equation 4) indicated a high degree of nucleophilic solvation to the ionization process, with *m* values of 0.95 (Equation 2) or 1.02 (Equation 4). The *h* value of 0.42 ± 0.15 obtained [61] with use of Equation 4 had only a 0.009 probability that the *hI* term is statistically insignificant, which suggested that the contribution from the *hI* term cannot be rejected [61,64]. The observations [58] that ethyl chloroformate (EtOCOCl) and ethyl thiochloroformate (EtSCOCl) proceeded by dual competing reaction channels that are heavily dependent on solvent ionizing ability coupled with evidence that MeOCOCl [65] followed an addition-elimination pathway in all solvents except in 90% 1,1,1,3,3,3-hexafluoro-2-propanol (HFIP), strongly suggest that more profound variations in the dominant mechanism may occur with the sulfur-for-oxygen substitution in ROCOCl [58–65]. Furthermore, it has been reported that the solvolysis of 2-thiophenecarbonyl chloride progresses in a unimolecular [66] fashion whereas the oxygen-for-sulfur substitution within the thiophene ring yields 2-furancarbonyl chloride which advances via a bimolecular addition-elimination process [67]. The present investigation continues to evaluate these trends seen in the solvolysis of thiochloroformate esters and we now report on the kinetics at 25.0 °C for the solvolyses of MeSCOCl (**1**) including those in solvents having an appreciable fluoroalcohol component.

## 2. Results and Discussion

The first-order specific rates of solvolysis for **1** were determined in 19 solvents at 25.0 °C. The solvents consisted of methanol (MeOH), ethanol (EtOH), and binary mixtures of water with methanol, ethanol, acetone, 2,2,2-trifluoroethanol (TFE), or HFIP, plus binary mixtures of TFE with ethanol. Additionally values in 100 EtOH and 80 EtOH were measured at 30.0 °C, values in 100 EtOH, 80 EtOH, 100 MeOH, 80 MeOH, and 90 HFIP were measured at 35.0 °C, and a value for 90 HFIP was also obtained at 45.0 °C. From literature values for the specific rates of solvolysis at several other temperatures, the Arrhenius Equation was used to calculate values at 25.0 °C for solvolyses in water [44]. The rate data for **1** in 20 solvents together with the literature values for *N*_T_ [7,8] and *Y*_Cl_ [4,68] are reported below in Table 1.

**Figure 2.**
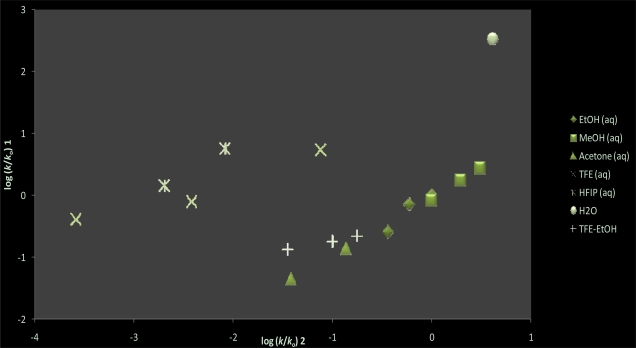
The plot of log (*k/k_o_*) for methyl chlorothioformate (**1**) against log (*k/k_o_*) for methyl chloroformate (**2**) in common pure and binary solvents.

**Figure 3.**
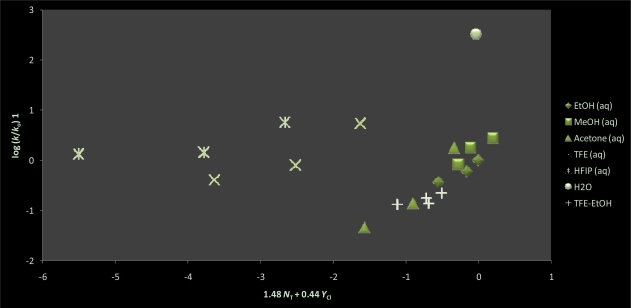
The plot of log (*k/k_o_*) for methyl chlorothioformate (**1**) against 1.48 *N*_T_ + 0.44 *Y*_Cl_.

In Table 1, the experimental first-order rate constants for the solvolysis of **1** increase as the proportion of water is increased in all the binary solvent mixtures (including fluoroalcohols) studied. These kinetic findings imply that mechanistically there is a strong dependence on solvent polarity. Using the rate data of the 20 solvents reported in Table 1, we report in Table 2 an extremely poor linear correlation using Equation 1, with *m* = 0.23 ± 0.06, *c* = −0.29 ± 0.16, 0.637 for the correlation coefficient, and 12 for the *F*-test value. This correlation improves marginally on use of the extended Grunwald-Winstein Equation (Equation 2) to lead to *l* = 0.64 ± 0.12, *m* = 0.60 ± 0.08, *c* = 0.10 ± 0.13, *R* = 0.879, and 29 for the *F*-test. Comparing the rates of **1** with those previously reported [69] for **2** and methyl fluoroformate (MeOCOF) in pure EtOH and MeOH at 35.0 °C, we observe a rate trend of *k***_1_** < *k***_2_** ≅ *k***_MeOCOF_**. The direction further shifts to *k***_1_** < *k***_2_** < *k***_MeOCOF_** in 80 EtOH at 35.0 °C. This signals that the inductive effect exerted by the methoxy group in **2** and in MeOCOF, makes the carbonyl carbon more positive and for the alcoholysis of **1**, a bimolecular type solvent mechanism may be consequential. As shown in Table 3, the extended Grunwald-Winstein Equation (Equation 2) has proven to be a powerful tool that has been used successfully to correlate the solvolyses of chloroformate [10,58,61,62,65,69] chlorothioformate [10,58,59], chlorothionoformate [10,60,61,63], and chlorodithioformate [10,60,61,64], esters, including instances where side-by-side mechanisms were under consideration. A plot of log (*k/k_o_*) for **1** against log (*k/k_o_*) for **2** in the common pure and binary solvents is shown in Figure 2. This plot validates the probability of dual competing reaction channels that is dependent on the ionizing ability of the solvent. As observed in Table 2, the extended Grunwald-Winstein analysis of the solvolysis of **1** is best carried out by dissecting the solvents based on solvent polarity. With 13 of the more nucleophilic solvents, we obtained a fair linear correlation with *l* = 1.47 ± 0.21, *m* = 0.49 ± 0.07, *c* = 0.14 ± 0.09, *R* = 0.927, and an *F*-test value of 30. Omitting the 60 acetone value, in 12 of the remaining nucleophilic solvents the correlation improves somewhat with *l* = 1.48 ± 0.18, *m* = 0.44 ± 0.06, *c* = 0.08 ± 0.08, *R* = 0.949, and an *F*-test value of 40. These values are very similar to the ones observed (Table 3) for the fluoro-, chloro-, and thio-chloroformate esters demonstrated [10,58–62,65,69] to solvolyze in the more nucleophilic solvents with rate-determining addition via an addition-elimination (association-dissociation) pathway. Reported in Table 1 are the activation parameters for **1** in 100 EtOH and 80 EtOH, in particular the negative value for the entropy of activation are compatible with those expected for a bimolecular process. Furthermore, the *l*/*m* ratio of 3.36 for **1** is analogous to the *l/m* ratio shown in Table 3 of PhSCOCl and PhOCSCl, which suggests that there exists a similar pattern of behavior between the specific rates of solvolysis in the more nucleophilic solvents in **1**, PhSCOCl and PhOCSCl. The plot of log (*k*/*k*_o_) **1** against 1.48 *N*_T_ + 0.44 *Y*_Cl_ shown in Figure 3 illustrates the two distinct mechanisms and advocates the possibility of a superimposed unimolecular pathway for 60 acetone. Using the Equation log (*k*/*k*_o_) = 1.44 *N*_T_ + 0.44 *Y*_Cl_ + 0.08, we calculate the specific rate by the bimolecular addition-elimination mechanism in 60 acetone to be 1.37 × 10^−5^. This signifies that **1** does solvolyze in 60 acetone by a dual pathway, viz., 32% bimolecular addition-elimination and 68% proceeds by an ionization mechanism.

In the highly ionizing aqueous fluoroalcohols, water, and 60 acetone, use of Equation 2 yields an excellent linear correlation with *l* = 0.79 ± 0.06, *m* = 0.85 ± 0.07, *c* = −0.27 ± 0.18, *R* = 0.987, and 95 for the *F*-test. These appreciable sensitivities towards changes in both solvent nucleophilicity and solvent ionizing power are similar to *l* = 0.83, and *m* = 0.70 observed for the solvolysis of acetyl chloride [41,57,70]; *l* = 0.69, and *m* = 0.95 for PhSCSCl [61]; and *l* = 0.66, and *m* = 0.93 for EtSCOCl [58], systems believed to follow an ionization pathway with appreciable nucleophilic solvation of the developing carbocation. It has been shown [41] that a theoretical G3 calculation for the gas phase heterolytic bond dissociation energy of **1** is 162.6 kcal/mol. This figure is almost identical to the value of 161.8 kcal/mol obtained for acetyl chloride [41] which attests to a similarity of heterolysis mechanisms for both substrates.

The slightly lower nucleophilic solvation requirement (*l* = 0.66) for EtSCOCl when compared to that in **1** (*l* = 0.79), is consistent with the additional carbon in EtSCOCl serving as a weak electron-donating substituent to stabilize the developing acylium ion. Additionally in most solvents studied (except in 100 EtOH, 100 MeOH, and 90 MeOH), EtSCOCl solvolyzes by a stepwise ionization mechanism with strong nucleophilic solvation (*l* = 0.66) of the developing acylium ion [58]. The negative entropy of activation (ΔS^≠^ = −28.4 ± 2.5 cal mol^−1^ K^−1^) observed in 90 HFIP as reported in Table 1 for **1**, further indicates a greater degree of ordering in the transition state than in the initial state which arises from the preferential tendency of strong rear-side solvation effects (association) at the developing carbocation.

At the other extreme, the photodecomposition of MeSCOCl (**1**) on irradiation with broad-band UV-visible light occurs in sequential steps [71] first forming CO and MeSCl. The authors then proposed a second step entailing detachment of a hydrogen atom from the methyl group of MeSCl with the formation of thioformaldehyde (H_2_C=S) and HCl [71].

## 3. Conclusions

The solvolysis of MeSCOCl (**1**) in the wide variety of solvents currently studied is found to be heavily dependent on the specific solvent properties of solvent nucleophilicity and solvent ionizing power. The empirical correlations presently carried out support the existence of concurrent side-by-side mechanisms, with a bimolecular association-dissociation (addition-elimination) mechanism favored in the more nucleophilic solvents and a stepwise ionization with profound rear-side nucleophilic solvation occurring in the highly ionizing binary solvent mixtures. The extended Grunwald-Winstein Equation (Equation 2) has once again proven to be an important empirical correlation tool that is sensitive enough to evaluate the relationships between reactivity and solute-solvent interactions.

## 4. Experimental Section

The *S*-methyl chlorothioformate (Sigma-Aldrich, 96%) was used as received. Solvents were purified and the kinetic runs carried out as described previously [72]. A substrate concentration of approximately 0.005 M in a variety of solvents was employed. For some of the runs, calculation of the specific rates of solvolysis (first-order rate coefficients) was carried out by a process in which the conventional Guggenheim treatment [73] was modified [74] so as to give an estimate of the infinity titer, which was then used to calculate for each run a series of integrated rate coefficients. The specific rates and associated standard deviations, as presented in Table 1, are obtained by averaging all of the values from, at least, duplicate runs.

Multiple regression analyses were carried out using the Excel 2007 package from the Microsoft Corporation, and the SigmaPlot 9.0 software version from Systat Software, Inc., San Jose, CA, was used for the Guggenheim treatments.

## Figures and Tables

**Table 1.** Specific rates of solvolysis (*k*) of **1**, in several binary solvents at 25.0 °C and literature values for (*N_T_*) and (*Y_Cl_*).

**Solvent (%)*a***	**1 @ 25.0 °C; 10^5^*k,* s^−1^*b***	***N*_*T*_*c***	***Y*_*Cl*_*d***
100% MeOH	2.00 ± 0.07*^e^*	0.17	−1.2
90% MeOH	4.29 ± 0.15	−0.01	−0.20
80% MeOH	6.75 ± 0.27*^f^*	−0.06	0.67
100% EtOH	0.884 ± 0.021*^g^*	0.37	−2.50
90% EtOH	1.45 ± 0.15	0.16	−0.90
80% EtOH	2.44 ± 0.12*^h^*	0.00	0.00
90% Acetone	0.107 ± 0.007	−0.35	−2.39
80% Acetone	0.334 ± 0.013	−0.37	−0.80
60% Acetone	4.30 ± 0.20	−0.52	1.00
97% TFE (w/w)	0.986 ± 0.030	−3.30	2.83
90% TFE (w/w)	1.92 ± 0.13	−2.55	2.85
70% TFE (w/w)	13.2 ± 1.5	−1.98	2.96
60T-40E	0.321 ± 0.015	−0.94	0.63
50T-50E	0.333 ± 0.017	−0.64	0.60
40T-60E	0.431 ± 0.013	−0.34	−0.48
20T-80E	0.537 ± 0.016	0.08	−1.42
100% H_2_O	820*^i^*	−1.38	4.57
97%HFIP (w/w)	3.21 ± 0.15	−5.26	5.17
90%HFIP (w/w)	3.48 ± 0.092*^j^*	−3.84	4.41
70%HFIP (w/w)	13.9 ± 0.78	−2.94	3.83

a Substrate concentration of *ca.* 0.0052 M; binary solvents on a volume-volume basis at 25.0 °C, except for TFE-H_2_O and HFIP-H_2_O solvents which are on a weight-weight basis. T-E are TFE-ethanol mixtures.

b With associated standard deviation.

c Refs. 7, 8.

d Refs. 4, 68.

e A value of 5.26 (± 0.03) × 10^−5^ s^−1^ was obtained at 35.0 °C.

f A value of 22.7 (± 1.02) × 10^−5^ s^−1^ was obtained at 35.0 °C.

g A value of 1.46 (± 0.18) × 10^−5^ s^−1^ and a value of 2.21 (± 0.12) × 10^−5^ s^−1^ was obtained at 30.0 °C and 35.0 °C respectively. A value 1.42 × 10^−5^ s^−1^ at 35.0 °C has been reported [48]. ΔH^≠^ = 15.0 ± 0.1 kcal/mol, ΔS^≠^ = −31.2 ± 0.3 cal mol^−1^ K^−1^.

h A value of 4.42 (± 0.14) × 10^−5^ s^−1^ and a value of 7.41 (± 0.16) × 10^−5^ s^−1^ was obtained at 30.0 °C and 35.0 °C respectively. ΔH^≠^ = 19.7 ± 0.6 kcal/mol, ΔS^≠^ = −13.5 ± 2.4 cal mol^−1^ K^−1^.

i Calculated from Arrhenius plots using the values at various temperatures reported in Ref. [44].

j A value of 7.72 (± 0.16) × 10^−5^ s^−1^ and a value of 18.4 (± 0.78) × 10^−5^ s^−1^ was obtained at 35.0 °C and 45.0 °C respectively. Δ H^≠^ = 15.1 ± 0.7 kcal/mol, ΔS^≠^ = −28.4 ± 2.5 cal mol^−1^ K^−1^.

**Table 2.** Correlation of the specific rates of reaction of **1** at 25.0 °C, using the simple or extended Grunwald-Winstein Equations (Equations 1 and 2).

**Substrate**	***n**a***	***l**b***	***m**b***	***c**c***	***R**d***	***F**e***
**1**	20*^f^*		0.23 ± 0.06	−0.29 ± 0.16	0.637	12
		0.64 ± 0.12	0.60 ± 0.08	0.10 ± 0.13	0.879	29
	13*g*		0.21 ± 0.13	−0.29 ± 0.16	0.435	3
		1.47 ± 0.21	0.49 ± 0.07	0.14 ± 0.09	0.927	30
	12*h*		0.17 ± 0.15	−0.34 ± 0.18	0.341	1
		1.48 ± 0.18	0.44 ± 0.06	0.08 ± 0.08	0.949	40
	8*i*		0.24 ± 0.26	−0.30 ± 0.97	0.341	1
		0.79 ± 0.06	0.85 ± 0.07	−0.27 ± 0.18	0.987	95

a Using data at 25.0 °C from Table 1; *n* is the number of solvents.

b With associated standard error.

c Accompanied by standard error of the estimate.

d Correlation coefficient.

e *F*-test value.

f All solvents.

g 100-80 EtOH-H_2_O, 100-80 MeOH-H_2_O, 90-60 Acetone-H_2_O, 60T-40E, 50T-50E, 40T-60E, 20T-80E.

h 100-80 EtOH-H_2_O, 100-80 MeOH-H_2_O, 90-80 Acetone-H_2_O, 60T-40E, 50T-50E, 40T-60E, 20T-80E.

i 97-70TFE-H_2_O, 97-70 HFIP-H_2_O, H_2_O, 60 Acetone-H_2_O.

**Table 3.** Correlation of the specific rates of reaction of other chloroformate and thiochloroformate esters using the extended Grunwald-Winstein Equation (Equation 2).

**Substrate**	***n**a***	***l**b***	***m**b***	***l/m***	***c**c***	***R**d***	***F**e***
EtOCOCl*f*	28	1.56 ± 0.09	0.55 ± 0.03	2.84	0.19 ± 0.24	0.967	179
	7	0.69 ± 0.13	0.82 ± 0.16	0.84	−2.40 ± 0.27	0.946	17
MeOCOCl*g*	19	1.59 ± 0.09	0.58 ± 0.05	2.74	0.16 ± 0.07	0.977	
PhOCOCl*h*	49	1.66 ± 0.05	0.56 ± 0.03	2.96	0.15 ± 0.07	0.980	568
PhSCSCl*i*	31	0.69 ± 0.05	0.95 ± 0.03	0.73	0.18 ± 0.05	0.987	521
PhOCSCl*j*	9	1.88 ± 0.28	0.56 ± 0.15	3.36	0.38 ± 0.15	0.950	28
	18	0.34 ± 0.05	0.93 ± 0.09	0.37	−2.54 ± 0.34	0.955	77
PhSCOCl*k*	16	1.74 ± 0.17	0.48 ± 0.07	3.63	0.19 ± 0.23	0.946	55
	6	0.62 ± 0.08	0.92 ± 0.11	0.67	−2.29 ± 0.13	0.983	44
EtSCOCl*l*	19	0.66 ± 0.08	0.93 ± 0.07	0.71	−0.16 ± 0.31	0.961	96
MeOCOF*m*	14	1.33 ± 0.09	0.73 ± 0.06	1.82	−0.08 ± 0.08	0.972	

a *n* is the number of solvents.

b With associated standard error.

c Accompanied by standard error of the estimate.

d Correlation coefficient.

e *F*-test value.

f Values taken from [58].

g Values taken from [65].

h Values taken from [61].

i Values taken from [61].

j Values taken from [61].

k Values taken from [60].

l Values taken from [58].

m Values taken from [69].
